# *Lactobacillus paracasei S16* Alleviates Lumbar Disc Herniation by Modulating Inflammation Response and Gut Microbiota

**DOI:** 10.3389/fnut.2021.701644

**Published:** 2021-08-10

**Authors:** Zhanchao Wang, Huiqiao Wu, Yu Chen, Huajiang Chen, Xinwei Wang, Wen Yuan

**Affiliations:** Department of Orthopaedics, Changzheng Hospital, Naval Medical University, Shanghai, China

**Keywords:** lumbar disc herniation, *Lactobacillus paracasei*, inflammation, gut microbiota, serum metabolomics

## Abstract

Lumbar disc herniation (LDH) is a common cause for low back pain. In this study, we aimed to explore the effects of a specific *Lactobacillus paracasei* (*L. paracasei*), *L. paracasei S16*, on the symptoms of LDH using a mouse model of LDH. The results showed that *L. paracasei S16* treatment improved the behavior, increased the cell proliferation, and decreased the apoptosis in LDH mice. Moreover, *L. paracasei S16* treatment alleviated the aberrant inflammation response in the LDH mice, which is characterized by the decreased anti-inflammatory cytokines, increased pro-inflammatory cytokines, and decreased percentage of Th1 and Th2 cells and Th17/Treg ratio. 16S rRNA sequencing results showed that the LDH mice treated with *L. paracasei S16* have higher relative abundance of *Lachnospiraceae* and *Ruminococcaceae* and lower abundance of *Lactobacillaceae* than mice in the LDH group. Additionally, the serum metabolites involved in the linoleic acid metabolism, alanine. aspartate, and glutamate, glycerophospholipid, and TCA cycle were significantly decreased and the metabolite involved in purine metabolism was significantly increased after the *L. paracasei S16* treatment in the LDH mice. These results showed that administration of *L. paracasei S16* can improve inflammation response, alter gut microbiota, and modulate serum metabolomics in a mouse model of LDH.

## Introduction

lumbar disc herniation (LDH) is one of the common spinal diseases and affects around 9% population worldwide (1). It has been well established that LDH is highly associated with the inflammation (2). For example, herniated disc tissue has increased levels of proinflammatory and regulatory cytokines, such as interleukin 1β (IL-1β), IL-4, IL-6, IL-12, tumor necrosis factor α (TNF-α), and interferon-γ (IFN-γ) (3–5). Further, these cytokines can activate the differentiation of lymphocyte. T helper 1 (Th1), Th2, and Th 17 lymphocytes plays an important role in activating inflammation, while Treg cell involves in preventing inflammation (6, 7). It has been demonstrated that patients with LDH have increased levels of circulating and disc Th17 and IL-17, which may contribute to pain (8).

Recent studies have shown that the *Lactobacillus paracasei* (*L. paracasei*) treatment can alleviate inflammation-related disorders by modulating the production of anti- and pro-inflammatory cytokines (9, 10). Furthermore, clinical studies also revealed the important role of *L. paracasei* supplementation in ameliorating inflammation in humans (11, 12). Mechanically, *L. paracasei* can act as a probiotic to improve gut microbial composition (13, 14). Increasing evidence has demonstrated that the gut microbiota is highly associated with the host inflammatory response. For example, the gut microbiota can influence the development of chronic inflammatory disorders by regulating the T cells function (15). However, whether the *L. paracasei* can alleviate aberrant inflammation in LDH mice by modulating the gut microbiota is unclear.

In this study, we investigated whether *L. paracasei* exert anti-inflammatory effects via modulating T cell function and gut microbiota in the mice with LDH. To test this hypothesis, we examined the effects of specific strain of *L. paracasei, L. paracasei S16*, on the behavior and the production of inflammatory cytokines in LDH mice. In addition, we also analyzed the gut microbiota and serum metabolomics to further explored the mechanism.

## Materials and Methods

### Reagents, Mice, and Ethics

The *Lactobacillus paracasei S16* was purchased from Hangzhou Hongsai biopharmaceutical Co., Ltd. (Zhejiang, China). The male Balb/C mice (20–15 g) were purchased from the Envigo (Indianapolis, USA). Mice were maintained in a 12 h-light/dark cycle and free access to diet and water. All procedures used in this experiment were approved by Changzheng Hospital Ethics Committee (No. 2020-0073).

### Mice and Surgery

The mice were divided into 4 groups (*n* = 12/group). The mice in the Sham and LDH groups were received 0.1 mL PBS, and the mice in the Sham + Probiotic and LDH + Probiotic groups were received 0.1 mL of 10^9^ CFU/ml *L. paracasei S16* via oral gavage for 4 weeks starting 1 week before the establishment of LDH. A LDH model was established as a previous study (16). Briefly, the mice were anesthetized with intraperitoneal injection of ketamine/xylazine. The lumbar 4-L (L4-L5) disc of mice in the Sham and Sham + Probiotic group were only exposed without puncturing laterally, while the L4-L5 dic of mice in the LDH and LDH +Probiotic groups were punctured laterally. At the post-operation day (POD) 28, blood samples were collected by orbital blooding. Serum was obtained by centrifugation of the blood samples at 1,000 g for 15 min under 4°C and stored in aliquots at −80°C.

### Measurement of Mechanical Allodynia and Thermal Hyperalgesia

The mechanical allodynia and thermal hyperalgesia were tested as reported previously (17). Briefly, the mechanical allodynia was measured by the incidence of foot withdrawal responding to non-noxious mechanical indentation of each hind paw using a probe with an 0.5 mm^2^ polypropylene tip. The thermal hyperalgesia was defined by the foot withdraw latency to heat stimulation.

### Immunohistochemistry

Dorsal root ganglia (DRG) samples were fixed in 4% paraformaldehyde overnight at 4°C. After embedding in paraffin, serial sections of 4 μm thickness were cut and treated with periodic acid to blocked the endogenous peroxidase. After incubated with the primary antibodies (Cyclin, Proteintech, USA; Ki67, Abcam, UK; PCNA, Proteintech, USA) at 4°C overnight, the sections were incubated with secondary antibodies for 30 min at 37°C. The images of the stained sections were captured by fluorescence microscope.

### Western Blot Analysis

The protein expression of Cyclin, Ki67, PCNA, Foxp3, IFN-γ, IL-2, IL-4, IL-5, IL-12, IL-17A, TGF-β, and IL-10 in the L4-L5 DRG were determined by Western blot (WB) analysis. Briefly, the samples were lysed in 0.1 mL lysis buffer and the lysate were centrifuged at 12,000 rpm for 15 min at 4°C. The proteins were transferred onto polyvinylidene difluoride membranes and blocked with 5% non-fat milk in tris-Tween-buffered saline buffer (20 mM tris, pH 7.5, 150 mM NaCl, and 0.1% Tween 20) for 1.5 hour and then incubated with the primary antibodies (Proteintech, USA; Abcam, UK) at 4°C overnight, followed by incubation with a goat anti-mouse IgG or a goat anti-rabbit IgG (Proteintech, USA) for 1 h at room temperature. Western blot bands were scanned and analyzed with Alpha Imager 2200 software (Alpha Innotech Corporation, CA, USA). Protein expression was normalized against β-actin.

### Terminal Deoxynucleotidyl Transferase Dutp Nick end Labeling (TUNEL) Assay

Cellular apoptosis was measured using the TUNEL assay kit according to the manufactures' instruction (Shanghai Yeasen biotech Co., Ltd., China).

### Flow Cytometric Analysis of T Cell Subsets

To determine the Th1, Th2, Th17, and Treg cells in mice, flow cytometric analysis was performed on isolated DRG cells using CD4, Foxp3, IL-17A, TGF-β, and IL-4 antibodies as reported previously (18). Briefly, the cells suspension was transferred into 1 mL phosphate buffer saline (PBS) and centrifuged at 350 g for 5 min. After centrifugation, the supernatant was removed and the cells were resuspended with 500 μL fixation/permeabilization then centrifuged at 350 g for 5 mice after standing at room temperature for 30 min. The resuspension was repeated for twice. The cells were then incubated with monoclonal antibodies, including CD4-FITC, FOXP3-PE, IL-17A-PE, IL-4-PE, and IFN-γ-PE antibodies (eBiosciences, San Diego, California, USA) at dark for 30 min. After washing with PBS, the cells were resuspended in 150 μL PBS and then tested by using Beckman counter flow cytometer (USA). The data were analyzed using FlowjoX software. The lymphocytes were gated by FSC and SSC. CD4^+^IL-17A^+^, CD4^+^IL-4^+^, CD4^+^ IFN-γ^+^, and CD4^+^ FOXP3^+^ lymphocytes were identified as Th17, Th2, Th1, and Treg respectively.

### Measurement of Serum Inflammatory Cytokines Levels

The levels of Foxp3, IFN-γ, IL-2, IL-4, IL-5, IL-12, IL-17A, TGF-β, and IL-10 in serum were measured by applying a manual enzyme-linked immunosorbent assay (ELISA)-based spectrophotometric approach involving the use of corresponding assay kits (Wuhan Huamei Bioengineering Co., Ltd, Wuhan, China).

### 16S rRNA Gene Sequencing

Fecal DNA was extracted using the QIAamp DNA Stool Mini Kit (Qiagen, Hilden, Germany) according to the manufacturer's instructions. DNA concentration and purity were monitored on 1% agarose gels. The V3-V4 region of the bacterial 16S ribosomal RNA gene was amplified using a specific primer (314F, 5′-CCTACGGGNGGCWGCAG-3′; 805R, 5′-GACTACHVGGGTATCTAATCC-3′). Amplicons were detected using 2% agarose gels electrophoresis and purified using the AxyPrep DNA gel extraction kit (Axygen Bioscience, CA, USA). After quantified and purified, paired-end sequencing was performed using an Illumina MiSeq instrument (Illumina, San Diego, CA, USA) at Shanghai Weihuan Bio-Pharm Technology Co. Ltd. (Shanghai, China) according to standard protocols. Raw sequencing data were deposited into the NCBI Sequence Read Archive (SRA) database associated with BioProject ID PRJNA729635. The sequences were analyzed and assigned to operational taxonomic units (OTUs; 97% identity), and chimeric sequences were identified and removed using UCHIME. Taxonomy was assigned to OTUs using the naïve Bayes classifier and q2-feature-classifier plugin against the SILVA-132-99 gene database, with a confidence threshold of 70%. Alpha diversity was analyzed using QIIME 2 (version 2.4), which included the calculation of observe, Chao1, ACE, Shannon, and Simpson indices. Beta diversity was estimated by computing the Bray-Curits distance among samples and visualized using Principal Co-ordinates Analysis (PCoA). The “VeenDiagram” package of R software and jvenn were used to produce Veen diagrams.

### Metabolomics Profiling of Serum Samples

The metabolomic process including sample preparation, metabolites extraction and detection, data processing and analysis. Briefly, 80 μL cold methanol was added to 20 μL serum. The mixture was vortexed for 1 min and then incubated at 4°C for 20 min. After centrifuging at 12,000 rpm for 10 min, the supernatant was collected, dried, and then resuspended for further analysis. A ACQUITY ultra-high-performance liquid chromatography system coupled to ABSciex Triple TOF 5600 (ABSciex, Franmingham, MA, USA) and an electrospray ionization source was used to tested the metabolomics profiling. Raw LC-MS data were analyzed using MarkerView and PeakView software for peak detection, identification, and alignment. Kyoto Encylopedia of Genes and Genomes (KEGG) database was used to identify the exact metabolites.

### Statistical Analysis

All statistical analyses were analyzed by one-way ANOVA followed by the Ducan test (SPSS 21 software). Data are expressed as the mean ± SEM. *P* < 0.05 was considered statically significant.

## Results

### *L. paracasei S16* Alleviated the Behavior in LDH Mice

**Figure 1:**
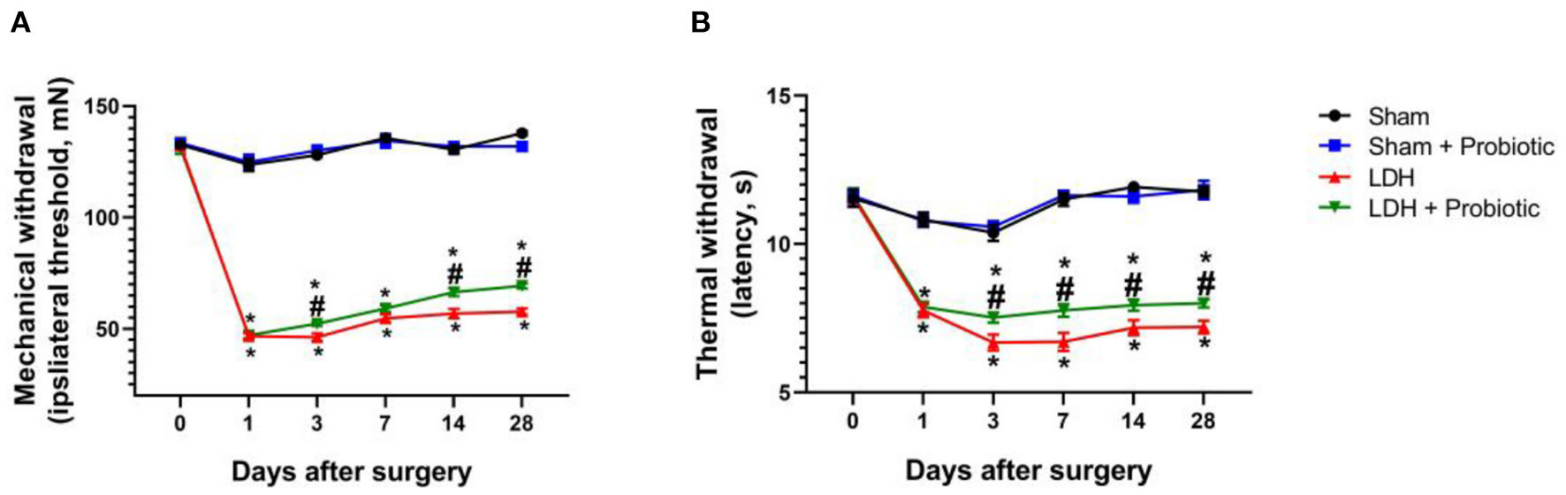
*L. paracasei S16* alleviated the behavior in LDH mice. **(A)** Mechanical withdrawal; **(B)** Thermal withdrawal. Data were expressed as the mean ± SEM. *P < 0.05 vs. Sham; ^#^*P* < 0.05 vs. LDH.

The mechanical allodynia and thermal hyperalgesia were tested to explore the effects of *L. paracasei S16* on behavior in LDH mice. The results showed that, in the LDH group, the mechanical and thermal withdraw were significantly decreased from the POD 1 to 28 compared with the Sham group (*P* < 0.05) (Figures 1A,B). However, the mechanical withdrawal at the POD 3, 14, and 28 and thermal withdraw from the POD 3 to 28 were significantly higher in the LDH + Probiotic group than the LDH group (*P* < 0.05) (Figures 1A,B), suggesting that *L. paracasei S16* treatment significantly alleviated the behavior of LDH mice.

### *L. paracasei S16* Elevated the Expression of Cell Proliferation Markers in LDH Mice

**Figure 2:**
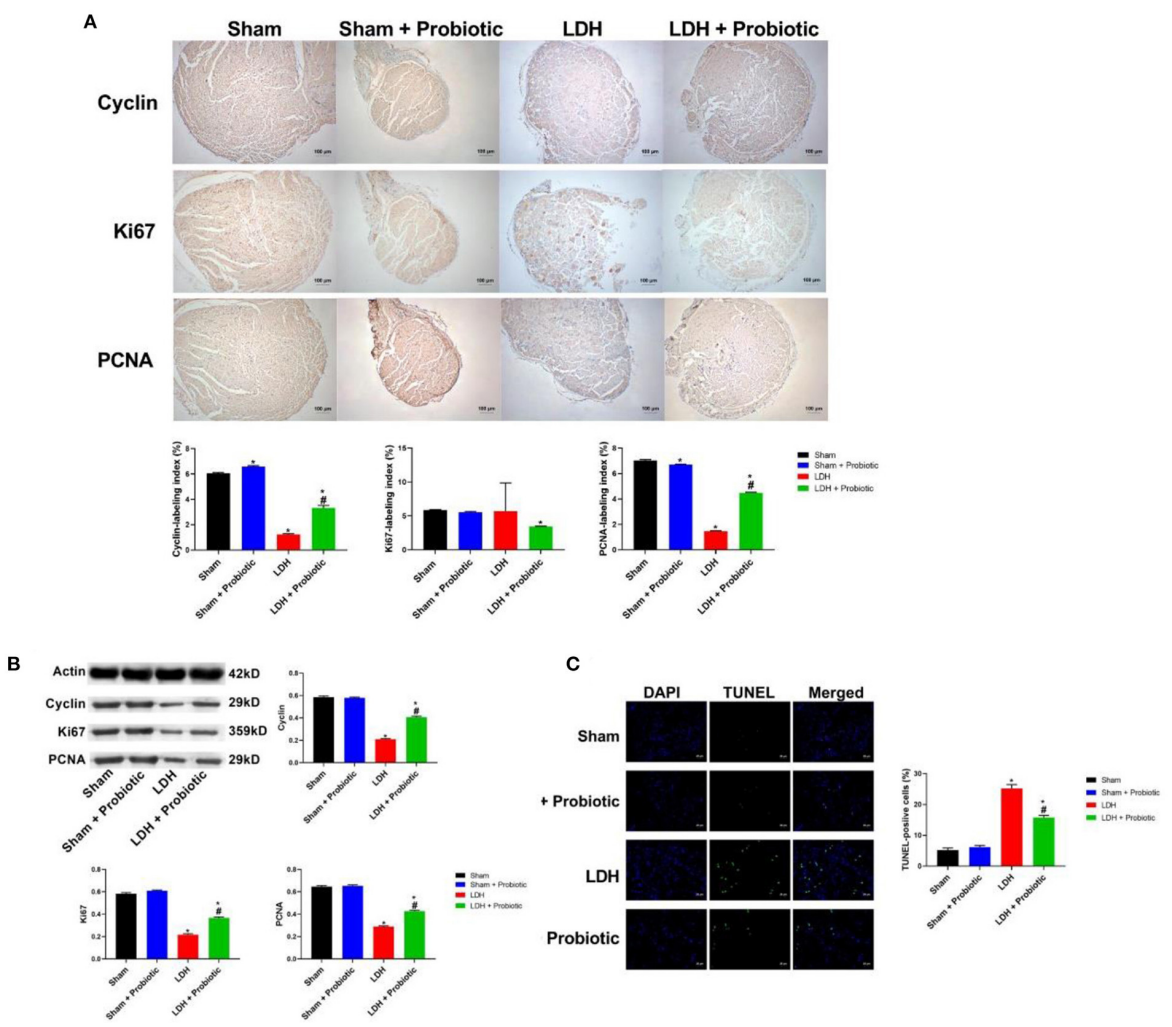
*L. paracasei S16* elevated the expression of cell proliferation markers in LDH mice. **(A)** Cyclin, Ki67, and PCNA protein expression in the DRG was examined by immunohistochemical staining; **(B)** Western blot analysis of cyclin, Ki67, and PCNA in DRG; **(C)** TUNEL analysis of DRG. Data were expressed as the mean ± SEM. *P < 0.05 vs. Sham; ^#^*P* < 0.05 vs. LDH.

We further examined the expressions of cell proliferation markers, Cyclin, Ki67, and PCNA in the DRG samples using IHC and WB. The IHC results showed that LDH mice have significantly lower expression of Cyclin and PCNA than the Sham mice, which was significantly reversed by the *L. paracasei S16* treatment (*P* < 0.05) (Figure 2A).

Similarly, the WB results showed that the LDH mice have significantly lower relative protein expression of Cyclin, Ki67, and PCNA (*P* < 0.05) (Figure 2B), suggesting that LDH mice have inhibited cell proliferation. However, *L. paracasei S16* treatment markedly increased the cell proliferation by enhancing the relative protein expressions of Cyclin, Ki67, and PCNA in LDH mice (*P* < 0.05) (Figure 2B).

The cellular apoptosis in DRG was measured by TUNEL assay. The results showed that the percentage of apoptotic to total cells was significantly higher in the LDH group than the Sham group, which was significantly reversed by the *L. paracasei S16* treatment (*P* < 0.05) (Figure 2C).

### *L. paracasei S16* Alleviated the Aberrant Inflammation in LDH Mice

**Figure 3:**
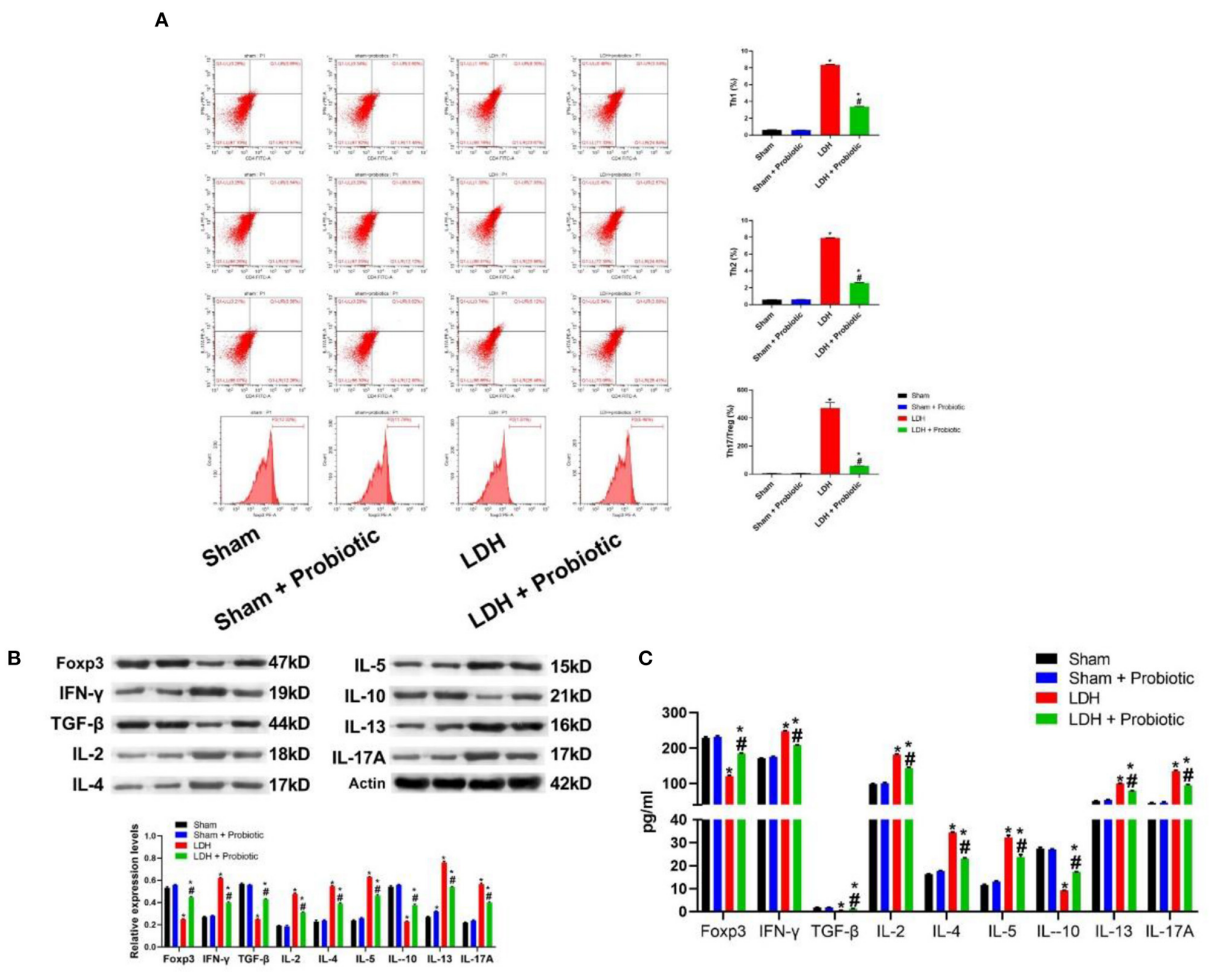
*L. paracasei S16* alleviated the aberrant inflammation in LDH mice. **(A)** Flow cytometric analysis of DRG; **(B)** Western blot analysis of inflammatory cytokines levels in the DRG; **(C)** ELISA of inflammatory cytokines in the serum. Data were expressed as the mean ± SEM. *P < 0.05 vs. Sham; ^#^*P* < 0.05 vs. LDH.

The comparisons of T cell subsets in the DRG were shown in Figure 3A. The percentage of Th1 and Th2 and the Th17/Treg ratio were significantly higher in the LDH group than the control group (*P* < 0.05). However, *L. paracasei S16* treatment significantly decreased the percentage of Th1 and Th2 and the Th17/Treg ratio in the LDH mice (*P* < 0.05).

Similarly, in the DRG, the relative protein expression levels of IFN-γ, IL-2, IL-4, IL-5, IL-12, IL-17A were significantly higher in the LDH group than the Sham group, which were significantly reversed by the *L. paracasei S16* treatment (*P* < 0.05) (Figure 3B). In addition, the relative protein expression levels of Foxp3, TGF-β and IL-10 were significantly lower in the LDH group than the Sham group, which were significantly reversed by the *L. paracasei S16* treatment (*P* < 0.05) (Figure 3B).

The serum levels of Foxp3, IFN-γ, IL-2, IL-4, IL-5, IL-12, IL-17A, TGF-β, and IL-10 were also measured using ELISA. The results showed that LDH mice have significantly higher levels of IFN-γ, IL-2, IL-4, IL-5, IL-12, and IL-17A, which were significantly reversed by the *L. paracasei S16* treatment (*P* < 0.05) (Figure 3C). In addition, the serum levels of Foxp3, TGF-β, and IL-10 were significantly lower in the LDH group than the Sham group, which were significantly reversed by the *L. paracasei S16* treatment (*P* < 0.05) (Figure 3C).

### *L. paracasei S16* Altered the Fecal Microbiota in LDH Mice

**Figure 4:**
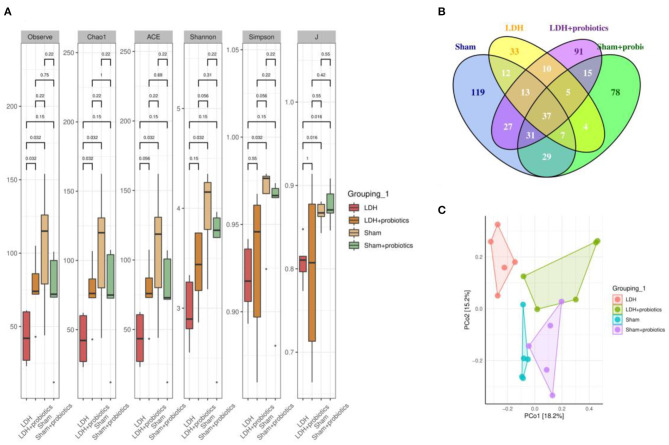
*L. paracasei S16* altered gut microbiota. **(A)** α-diversity; **(B)** Venn diagram; **(C)** PCoA. Data were expressed as the mean ± SEM.

The fecal microflora was analyzed by sequencing V3+V4 regions of 16S rRNA genes. To identify the microbial α-diversity, Observe, Chao1, ACE, Shannon, Simpson, and J indexes were examined. As shown in Figure 4A, LDH mice had significantly lower α-diversity than the Sham mice, which is characterized by the decreased Observe, Chao1, ACE, Shannon, Simpson, and J indexes (*P* < 0.05) (Figure 4A). However, *L. paracasei S16* treatment significantly increased the α-diversity in the LDH mice by increasing the Observe, Chao1, ACE, Shannon, Simpson, and J indexes (*P* < 0.05) (Figure 4A). The Venn diagram showed that there are 37 common OTUs between the four groups. Meantime, the Sham, Sham + Probiotic, LDH, LDH + Probiotic mice contained individual 119, 33, 91, and 78 OTUs, respectively (Figure 4B). To further understand the microbial composition between the two groups, we evaluated beta-diversity using PCoA based on Bray-Curtis distance. The results showed that the microbial community structure in the four groups were significantly different (Figure 4C).

**Figure 5:**
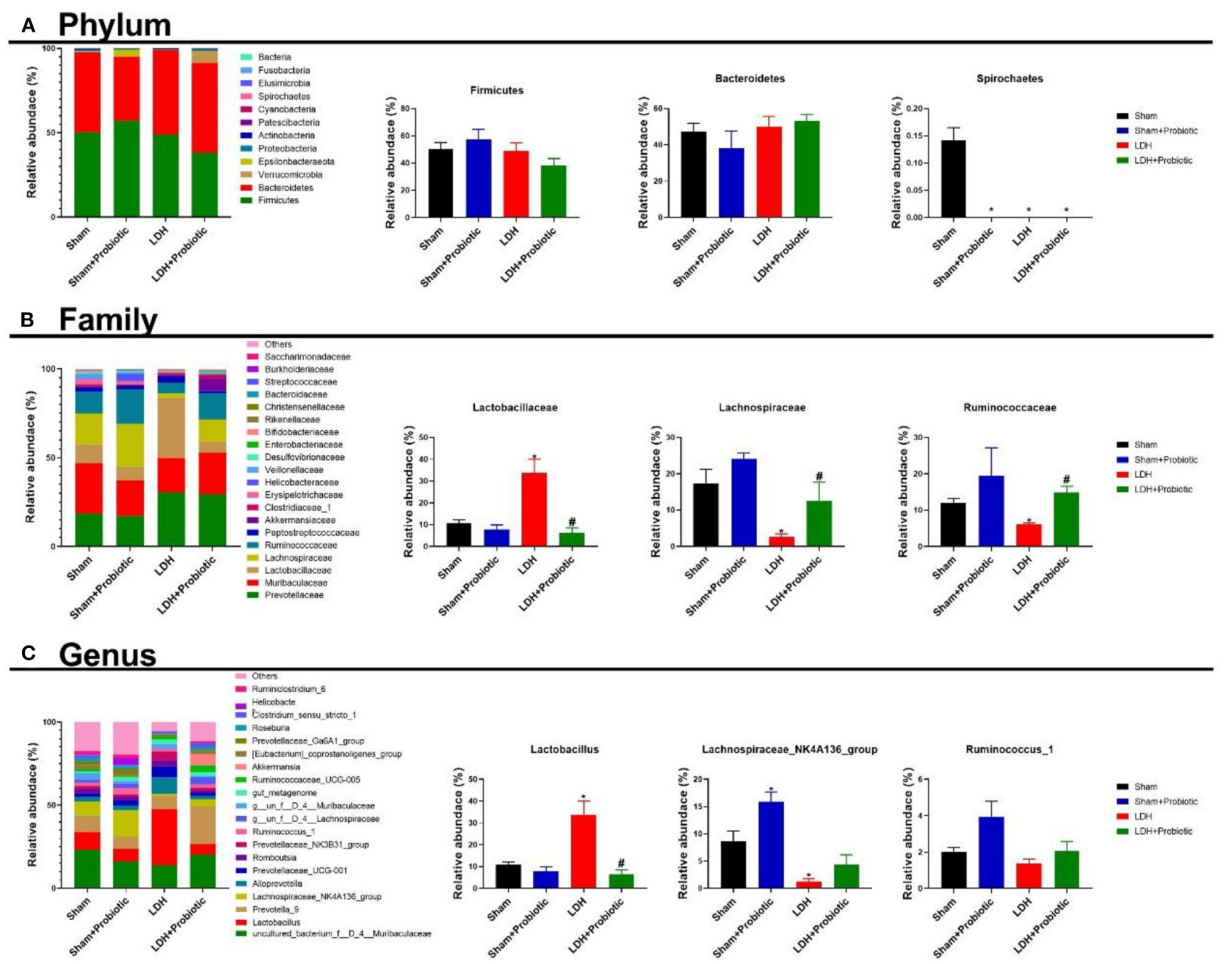
Fecal microbial composition. **(A)** Phylum level; **(B)** Family level; **(C)** Genus level. Data were expressed as the mean ± SEM. *P < 0.05 vs. Sham; ^#^*P* < 0.05 vs. LDH.

We further analyzed the microbial compositions at the phylum and family levels. As shown in Figure 5A, at the phylum level, Sham mice had significantly higher relative abundance of *Spirochaetes* than the other three groups (*P* < 0.05). At the family level, the LDH mice had significantly higher relative abundance of *Lactobacillaceae* than the Sham mice, which was significantly reversed by the *L. paracasei S16* treatment (*P* < 0.05) (Figure 5B). Meantime, the relative abundance of *Lachnospiraceae* and *Ruminococcaceae* are significantly lower in the LDH group than the Sham group (*P* < 0.05) (Figure 5B). However, *L. paracasei S16* treatment significantly increased the relative abundance of *Lachnospiraceae* and *Ruminococcaceae* in the LDH mice (*P* < 0.05) (Figure 5C). At the genus level, LDH mice have significantly higher relative abundance of *Lactobacillus* and lower relative abundance of *Lachnospiraceae_NK4A136_group* than the control mice (*P* < 0.05) (Figure 5C). Interestingly, *L. paracasei S16* supplementation significantly decreased the relative abundance of *Lactobacillus* in mice with LDH (*P* < 0.05) (Figure 5C). Additionally, *L. paracasei S16* significantly enhanced the relative abundance of *Lachnospiraceae_NK4A136_group* in the sham mice (*P* < 0.05) (Figure 5C).

### *L. paracasei S16* Altered the Serum Metabolomics in LDH Mice

**Figure 6:**
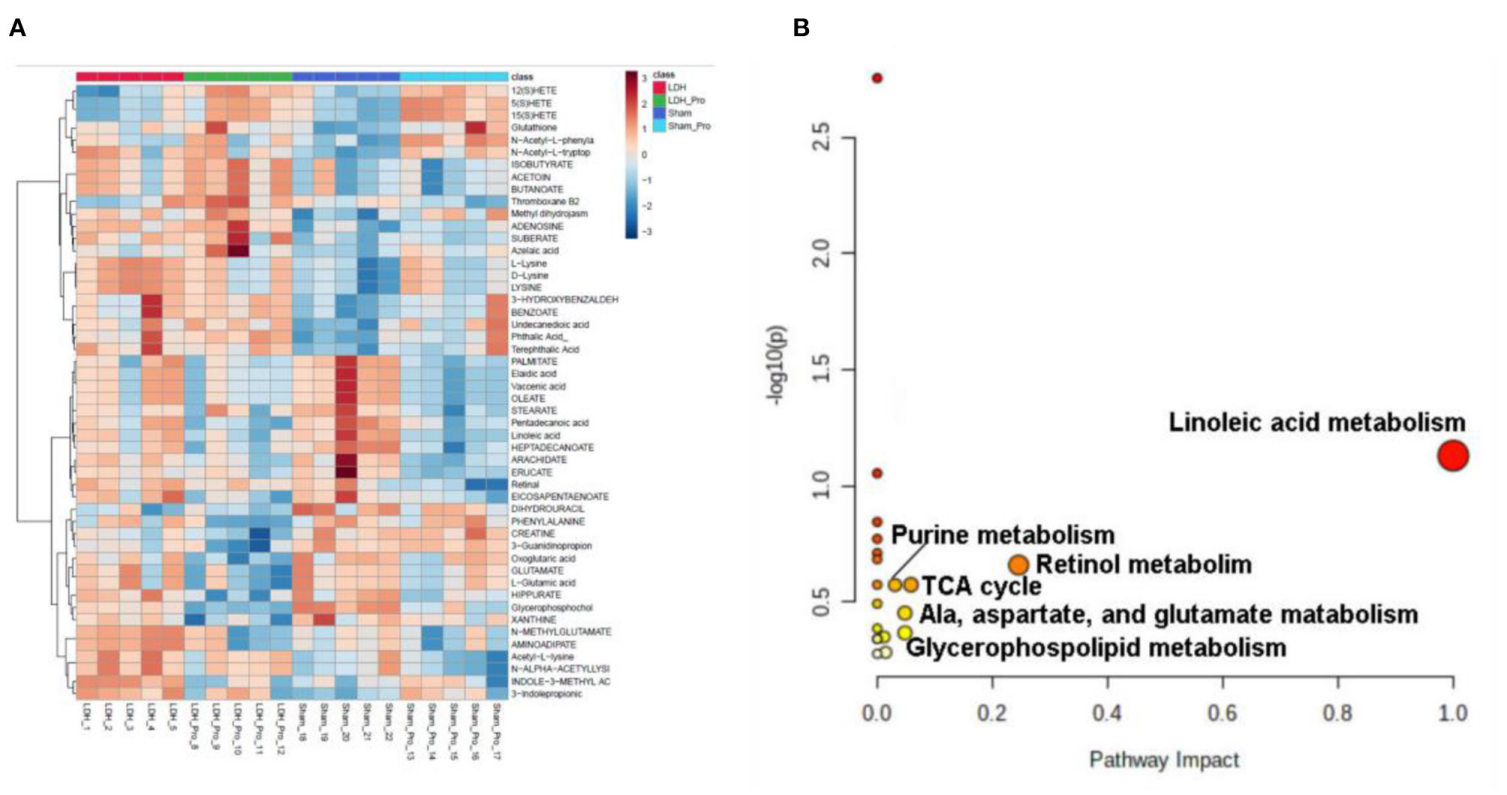
*L. paracasei S16* changed serum metabolomics changes. **(A)** Heat-map of the intensity of 32 significantly different metabolites showing significantly different metabolic profiles between the Sham, Sham + Probiotic, LDH, LDH + Probiotic groups. **(B)** The disturbed metabolic pathways showed differential metabolites by MetaboAnalyst 5.0 software. Node radius was based on pathway impact values. Node color was based on *P* value.

Serum metabolomics were examined to explore the metabolites altered by *L. paracasei* S16 treatment. The results showed that, there were 32 differential metabolites in the four groups (Figure 6A). The potential metabolic pathways of the differential metabolites were analyzed using MetaboAnalyst 5.0 software. The results showed that the differential metabolites involved in linoleic acid metabolism, Retinol metabolism, Alanine, aspartate and glutamate metabolism, Glycerophospholipid metabolism, citrate cycle (TCA cycle), and purine metabolism (Figure 6B).

## Discussion

LDH is one of the most common cause of low back pain. In this study, we investigated the effects of *L. paracasei S16* administration on the symptoms of LDH in a mouse model. Our results demonstrated that the supplementation with a specific *L. paracasei* strain, *L. paracasei S16*, could ameliorate the symptoms of LDH through improving inflammation, modulating gut microbiota, and altering serum metabolites.

The results of behavior test showed that *L. paracasei S16* treatment had alleviating action on LDH mice, which is characterized by the increased mechanical withdrawal and thermal withdraw. It has been reported that LDH exhibited increased cell apoptosis and decreased cell proliferation (19, 20). Thus, in this study, we examined the effects of *L. paracasei S16* treatment on the expressions of cell proliferation-related markers. The results showed that *L. paracasei S16* treatment increased the relative protein expressions of Cyclin, Ki67, and PCNA in LDH mice and decreased the apoptosis, suggesting that *L. paracasei S16* may alleviate LDH by inhibiting apoptosis and promote cell proliferation in DRG tissue.

Numerous studies have shown that LDH is accompanied by disordered inflammatory responses, such as increased production of pro-inflammatory cytokines and decreased anti-inflammatory cytokines (3–5). Consistently, in this study, we found that LDH mice have higher levels of pro-inflammation cytokines (IFN-γ, IL-2, IL-4, IL-5, IL-12, and IL-17A) and lower levels of anti-inflammatory cytokines (Foxp3, TGF-β, and IL-10) in the serum and DRG tissue. However, *L. paracasei S16* treatment decreased the production of pro-inflammatory cytokines and increased the production of anti-inflammatory cytokines in the mice with LDH. Similarly, previous studies also found that *L. paracasei* can modulate inflammation by decreasing the production of proinflammatory cytokines (IL-1, IL-2, and TNF-α) and increasing the production of anti-inflammatory cytokines (IL-10 and TGF-β) production and inhibiting inflammatory activation (9, 10). These data showed that *L. paracasei S16* treatment may alleviate LDH by improve inflammation in mice.

Activated T cells can differentiate into different subsets, including Th1, Th2, Th17, and Treg cells, that contribute to immune response. Th1 and Th2 cells can produce IFN-γ and IL-4, IL-13, and IL-5, respectively. Th17 has been shown to play an important role in inducing inflammation and autoimmune diseases (including LDH) by secreting its effector cytokine, IL-17 (6). Contrarily, Treg cells can prevent autoimmunity. TGF-β and Foxp3 are involved in the differentiation Th17 cells and Treg cells, respectively (7). Furthermore, the imbalance of Th17/Treg ratio can cause autoimmune disorders (7, 21). In this study, the data on T cell subsets showed that LDH is associated with higher levels of Th1, Th2, and Th17/Treg ration, which were alleviated by the *L. paracasei s16* treatment. Collectively, these data suggested that *L. paracasei s16* treatment can alleviate inflammation by influencing the production of inflammatory cytokines and the differentiation of T cells.

Amounting studies have demonstrated that gut microbiota plays an important role in modulating inflammatory response. We found that, compared to the sham mice, the LDH mice have decreased relative abundance of *Spirochaetes*, which is the most neurotropic bacteria (22). However, it has been proved that *Spirochaetes* can induce the production of pro-inflammatory cytokines and cause chronic inflammation (23, 24). Furthermore, animal and clinical studies showed that *L. paracasei* can improve inflammation by modulating gut microbiota (25, 26). Contrarily, in this study LDH mice had higher abundance of *Lactobacillaceae* than the Sham mice. Administration with *L. paracasei s16* decreased the abundance of *Lactobacillaceae* in mice with LDH. Similarly, at the genus level, *L. paracasei s16* decreased the relative abundance of *Lactobacillus*. These conflicting results need further investigation. Additionally, *L. paracasei s16* administration increased the relative abundance of *Lachnospiraceae* and *Ruminococcaceae* in mice with LDH. It has been reported that the short chain fatty acids, such as butyrate and propionate, produced by *Lachnospiraceae* involved in activating Treg cells, reducing pro-inflammatory cytokines, and increasing anti-inflammatory cytokines, which collectively alleviate inflammation (27–29). Similarly, *Ruminococcaceae*, which increased after *L. paracasei s16* administration in mice with LDH, can ameliorate chronic inflammation by producing butyrate (29). Thus, in this study, we speculated that *L. paracasei s16* treatment regulated the T cells populations by modulating gut microbiota, which contribute to alleviating aberrant inflammatory response.

In addition, we also examined the serum metabolomics to explore whether *L. paracasei s16* supplementation improve inflammation through changing serum metabolites in LDH mice. The data showed that the metabolites involved in the linoleic acid metabolism (linoleate), alanine. aspartate, and glutamate (oxoglutaric acid), glycerophospholipid (glycerophosphocholine), and TCA cycle (oxoglutaric acid) were significantly decreased and the metabolite involved in purine metabolism (adenosine) was significantly increased after the *L. paracasei S16* treatment in LDH mice. Similarly, previous studies also found that linoleic acid plays an important role in promoting inflammation (30). A recent clinical study analyzing the relationship between serum metabolites and inflammation showed that the serum oxoglutaric acid has a negative correlation with the inflammation severity. However, in this study, we found that *L. paracasei S16* treatment decreased the serum level of oxoglutaric acid, which may be because the different animal models. Thus, although the specific mechanism is unclear, it is reasonable to hypothesize that *L. paracasei S16* treatment may improve inflammation by modulating the serum metabolites.

Taken together, the current study demonstrated that *L. paracasei S16* treatment can alleviate inflammation by modulating serum metabolites and gut microbiota in LDH mice. However, the casual role of altered gut microbiota in the suppressed inflammation and changes in serum metabolites need further confirmation.

## Data Availability Statement

The datasets presented in this study can be found in online repositories. The names of the repository/repositories and accession number(s) can be found here: https://www.ncbi.nlm.nih.gov/Traces/study/?acc=PRJNA729635.

## Ethics Statement

The animal study was reviewed and approved by Changzheng Hospital Ethics Committee (No. 2020-0073).

## Author Contributions

ZW, HC, and XW designed this study. ZW, HW, and YC participated in the experiment. ZW, HC, and WY analyzed the experiment data. HW and YC wrote and revised this manuscript. All the authors read the final manuscript and agreed to publish it.

## Conflict of Interest

The authors declare that the research was conducted in the absence of any commercial or financial relationships that could be construed as a potential conflict of interest.

## Publisher's Note

All claims expressed in this article are solely those of the authors and do not necessarily represent those of their affiliated organizations, or those of the publisher, the editors and the reviewers. Any product that may be evaluated in this article, or claim that may be made by its manufacturer, is not guaranteed or endorsed by the publisher.
